# Rapid and accurate species identification for ecological studies and monitoring using CRISPR‐based SHERLOCK

**DOI:** 10.1111/1755-0998.13186

**Published:** 2020-06-13

**Authors:** Melinda R. Baerwald, Alisha M. Goodbla, Raman P. Nagarajan, Jonathan S. Gootenberg, Omar O. Abudayyeh, Feng Zhang, Andrea D. Schreier

**Affiliations:** ^1^ California Department of Water Resources Sacramento CA USA; ^2^ Department of Animal Science University of California Davis Davis CA USA; ^3^ Broad Institute of the Massachusetts Institute of Technology (MIT) and Harvard Cambridge MA USA; ^4^ McGovern Institute for Brain Research MIT Cambridge MA USA; ^5^ Department of Brain and Cognitive Science MIT Cambridge MA USA; ^6^ Department of Biological Engineering MIT Cambridge MA USA; ^7^ Department of Systems Biology Harvard University Boston MA USA; ^8^ Department of Health Sciences and Technology MIT Cambridge MA USA

**Keywords:** Cas13a, DNA extraction‐free, field‐based diagnostic, genetic species identification, noninvasive sampling, Osmerid smelt

## Abstract

One of the most fundamental aspects of ecological research and monitoring is accurate species identification, but cryptic speciation and observer error can confound phenotype‐based identification. The CRISPR‐Cas toolkit has facilitated remarkable advances in many scientific disciplines, but the fields of ecology and conservation biology have yet to fully embrace this powerful technology. The recently developed CRISPR‐Cas13a platform SHERLOCK (Specific High‐sensitivity Enzymatic Reporter unLOCKing) enables highly accurate taxonomic identification and has all the characteristics needed to transition to ecological and environmental disciplines. Here we conducted a series of “proof of principle” experiments to characterize SHERLOCK’s ability to accurately, sensitively and rapidly distinguish three fish species of management interest co‐occurring in the San Francisco Estuary that are easily misidentified in the field. We improved SHERLOCK’s ease of field deployment by combining the previously demonstrated rapid isothermal amplification and CRISPR genetic identification with a minimally invasive and extraction‐free DNA collection protocol, as well as the option of instrument‐free lateral flow detection. This approach opens the door for redefining how, where and by whom genetic identifications occur in the future.

## 1 INTRODUCTION

Accurate species identification is essential for ecological research and environmental monitoring. Some species are easy to identify visually, while identification of others is more challenging due to cryptic speciation (Hubert et al., 2008) and phenotypic plasticity (Pinzón et al., 2013). In these cases, as well as for more refined taxonomic discrimination (e.g., populations), genetic methods are often considerably more accurate (Benjamin et al., 2018; Vrijenhoek, 2009). To date, genetic identification has required a trained geneticist to receive the sample, conduct molecular methods (usually in a laboratory), analyse results and report the findings back to their field collaborators. This process can require days, and possibly even months, thus delaying the progression of research, conservation, and management actions based on the findings. In addition, laboratory facilities may not be available for genetic species identification in remote field locations or in countries with developing scientific infrastructure. A field‐deployable genetic‐based approach would allow biologists to quickly identify species in the field. Rapid species identification ensures accurate data collection in ecological studies and can be critically important for time‐sensitive species management and compliance with laws protecting threatened species. For example, human activities (e.g., logging, fishing, sample collection) that may jeopardize a protected species must be rapidly altered or stopped once the number of individuals permitted to be “taken” by this activity under U.S. Endangered Species Act regulations has been met. Field‐deployable identification will allow scientists working in remote locations and developing nations to conduct their own genetic species identification in situ. Customs agents and wildlife forensics specialists could also benefit from rapid species identification at border crossings or crime scenes, respectively. CRISPR (clustered regularly interspaced short palindromic repeats)‐based genetic methods could be ideal for species identification, due to their diagnostic specificity, sensitivity and speed (Knott & Doudna, 2018). The recently developed CRISPR‐based SHERLOCK nucleic acid detection platform has shown promise in the fields of diagnostic healthcare (Gootenberg et al., 2017, 2018; Myhrvold et al., 2018) and agriculture (Abudayyeh, Gootenberg, Kellner, & Zhang, 2019). SHERLOCK combines isothermal amplification with the functional capability of Cas13a to indiscriminately cleave RNA (including reporter RNA) only after it detects a specific target sequence. Many of the qualities that make it attractive for field deployment in healthcare and agriculture (e.g., rapid detection, single temperature reaction condition, high sensitivity, low cost) make it well suited to transition to an ecological context.

**Figure 1:**
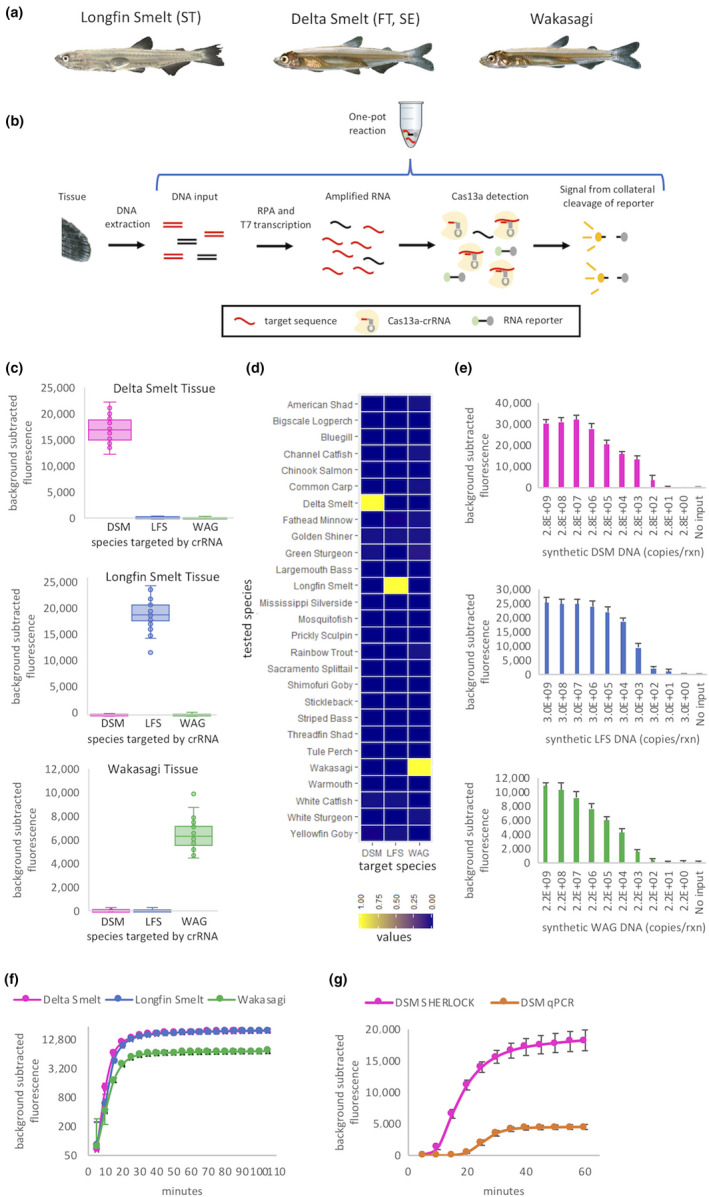
Accurate, sensitive and rapid species‐specific diagnostics using DNA from tissue with the SHERLOCK platform. DSM = delta smelt, LFS = longfin smelt, WAG = wakasagi. (a) Phenotypic comparison of three osmerid fish species (juvenile life stage) targeted for SHERLOCK differentiation (Photo credit: Rene C. Reyes). ST = listed as threatened by state of California; SE = listed as endangered by state of California; FT = listed as threatened by USA. (b) SHERLOCK schematic. DNA is extracted from a small amount of tissue. In a single reaction, the DNA is converted to amplified target RNA that binds to target‐specific crRNA. Activated Cas13a then collaterally cleaves an RNA reporter, causing fluorescence. (c) Osmerid specificity based on fluorescence after 1 hr. DNA from each target species was tested against all three species‐specific crRNAs. *N* = 40 biological replicates for target species and *N* = 20 biological replicates for nontarget species for each crRNA. Boxplots display the median and interquartile range for each DNA and crRNA assay combination. (d) Species‐specific identification with each column representing a crRNA and each row representing a common fish species found in the San Francisco Estuary. Fluorescence values are the background‐subtracted average from two biological replicates per species followed by normalization for each smelt species assay. (e) Limit of detection for each SHERLOCK assay using species‐specific crRNA and serial dilutions of species DNA derived from a synthetic template. Fluorescence was measured after 1 hr and bars represent means (± 1 *SD*) from three technical replicates. (f) SHERLOCK time‐course. Fluorescence of species‐specific crRNA combined with 20 ng DNA from each target species. Fluorescence was measured every 5 min over a 110‐min time‐course. Three biological replicates were averaged per species (± 1 *SD*). (g) Comparison of delta smelt SHERLOCK and qPCR time‐course. SHERLOCK conditions and results are same as in (f). The qPCR also used 20 ng DNA as template and amplified the same *cyt‐b* region as SHERLOCK by using a TaqMan assay. Fluorescence was measured every 5 min over a 1‐hr time‐course. Three biological replicates were averaged (± 1 *SD*).

In this study, as a proof of principle, we engineered SHERLOCK DNA assays that do not require DNA extraction or specialized equipment to genetically distinguish three morphologically similar fish species (Figure 1a) with range overlap in California's San Francisco Estuary (SFE) (Figure S1). Specifically, we sought to reliably distinguish the US threatened and California endangered delta smelt (DSM; *Hypomesus transpacificus*) (CDFW, 2019; USFWS, 1993), the California threatened longfin smelt (LFS; *Spirinchus thaleichthys*) (CDFW, 2019) and the non‐native wakasagi (WAG; *Hypomesus nipponensis)*. All three are members of the family Osmeridae and are particularly difficult to distinguish morphologically at younger life stages. For example, a recent study found substantial morphology‐based field misidentifications between juvenile delta smelt and wakasagi (Benjamin et al., 2018). Field‐deployable genetic assays for these species would enable real‐time decision‐making when evaluating protected species “take.” Real‐time knowledge of take would benefit the year‐round ecological monitoring programmes occurring throughout the SFE as well as state and federal water export facilities, which are required to substantially scale back exports when take limits for protected species are exceeded. More generally, a SHERLOCK‐enabled field taxonomic identification method could be broadly utilized by nonmolecular biologists working in the fields of ecology, conservation biology and environmental monitoring for any target species.

## 2 MATERIALS AND METHODS

### 2.1 Production of LwCas13a, crRNAs and gBlock fragments

LwCas13a protein was synthesized and purified by GenScript. Guide CRISPR RNAs (crRNAs) were synthesized by Integrated DNA Technologies (IDT) as ultramer RNA and rehydrated following Dharmacon's synthetic guide RNA resuspension protocol (Dharmacon). Gene block (gBlock) fragments were synthesized by IDT for use in sensitivity and qPCR reactions.

### 2.2 RPA primer and crRNA design

We used published mitochondrial cytochrome b (*cyt‐b*) sequences for DSM, LFS and WAG (Baerwald, Schumer, Schreier, & May, 2011; Brandl et al., 2015) to identify diagnostic polymorphisms between the species for recombinase polymerase amplification (RPA) primer design (Figure 1b; Figure S2, Tables S1‐S3). Sequences were downloaded from NCBI and then aligned in mega7 (Kumar, Stecher, & Tamura, 2016). RPA primers for delta smelt were taken directly from Baerwald et al. (2011) while new primers were designed for both longfin smelt and wakasagi (Table S1) using primer3web version 4.1.0 (Koressaar & Remm, 2007; Untergasser et al., 2012). Forward primers contained the T7 promoter sequence (TAATACGACTCACTATAGGG) at the 5′ end along with four or five additional bases to increase binding affinity.

The crRNAs were designed following the guidelines in Gootenberg at al. (2017). Each crRNA was 67 nucleotides in length with a 28‐nucleotide spacer sequence and contained the T7 binding sequence (Table S2). None of our protospacer flanking sites (PFS) for the smelt species contained G, which reduces LwCas13a cleavage robustness. We introduced a mismatch in position 5 of the spacer to increase the specificity of LwCas13a (Gootenberg et al., 2017). The Multiple Primer Analyzer tool (ThermoFisher Scientific) was used to ensure that both RPA primers and crRNAs that formed self‐dimers or cross‐primer dimers were not taken through to production.

### 2.3 DNA extraction from tissue

DNA was extracted from a 2 × 2‐mm caudal fin tissue piece using the DNeasy Blood and Tissue kit (Qiagen). We followed the manufacturer's protocol with a few modifications. Dissected fin was incubated at 56°C overnight (~16 hr) in Buffer ATL and Proteinase K solution. Samples were eluted in 100 µl of DNase/RNase‐free water. DNA concentration was measured using the Qubit dsDNA Broad Range Assay Kit read on the Qubit 2.0 (Life Technologies).

### 2.4 SHERLOCK assay

Detection reactions were performed as described in Gootenberg et al. (2017) with a few modifications. Briefly, a single reaction assay consisted of 0.48 µm forward primer, 0.48 µm reverse primer, 1 × RPA rehydration buffer (TwistDx), varying amounts of DNA input, 45 nm LwCas13a recombinant protein, 22.5 nm crRNA, 200 nm substrate reporter (RNaseAlert‐1 Substrate, IDT), 4 µl murine RNase inhibitor (New England Biolabs), 2 mm ATP, 2 mm GTP, 2 mm UTP, 2 mm CTP (New England Biolabs), 1 µl T7 polymerase mix (New England Biolabs), 5 mm MgCl_2_ (Invitrogen) and 14 mm MgAc (TwistDx). The addition of background RNA was excluded. Initial testing of primer pairs using only RPA was bypassed and we started directly with the combined collateral detection reaction (RPA with LwCas13a, crRNA and reporter).

RPA amplification (TwistAmp Basic RPA kit, TwistDx) occurred in a total volume of 12 µl (excluding DNA input). Two microlitres of DNA input was added to each reaction. Reactions were set up in a laminar flow hood to reduce the chance of contamination. Reactions were carried out in BioRad white shell qPCR plates and then incubated at 37°C for 1 hr and 45 min with fluorescent plate readings every 5 min for a total of 21 cycles. Fluorescent excitation and emissions were measured using the FAM channel on the BioRad CFX96 Touch Real‐Time PCR Detection System (BioRad). BioRad CFX Maestro Software was used to obtain relative fluorescent units for each sample across cycles. Duplicate negative control samples were included on each plate and their average was used to background‐subtract all samples for each cycle.

An initial assay screen was completed using DNA extracted from the caudal fin of two target individuals and one of each of the nontarget species. For example, delta smelt reactions were tested with two delta smelt individuals and one each of longfin smelt and wakasagi. For additional screening, we selected highly specific crRNA/RPA primer pair combinations with the greatest fluorescence intensity and most rapid amplification (one pair per species).

### 2.5 Tissue specificity reactions

The original *cyt‐b* TaqMan assays designed to distinguish delta smelt, longfin smelt and wakasagi were extremely specific and did not display any signs of cross‐amplification (Baerwald et al., 2011; Brandl et al., 2015). We conducted additional screening of our best crRNA/RPA primer pairs (one per species) to see if they exhibited similar specificity. Background‐subtracted fluorescence for 40 target fish and 20 each of the other nontarget smelt species were assessed for each of the three osmerid SHERLOCK reactions after 1 hr at 37°C. Boxplots were used to visualize the median and interquartile range for each DNA and crRNA assay combination.

Additionally, we screened two individuals from each of the 24 nontarget fish species (Table S4) found throughout the same geographical range as all three smelts (Figure S1). These 48 samples, along with target smelt samples, were run with all three individual smelt assays to ensure specificity. Fluorescence values for the biological replicates were background‐subtracted, averaged and then normalized based on the highest fluorescence values across all species after 1 hr at 37°C. These normalized values were graphically displayed by creating a heatmap using ggplot2 in R (R Core Team, 2019).

### 2.6 gBlock sensitivity reactions

gBlocks were synthesized by IDT for all three of our target species. The gBlocks contained the *cyt‐b* amplified region with 20 additional flanking bases on either end and the T7 promoter sequence on the 5′ end. Serial dilutions starting with between 2.2 and 3 billion copies per reaction were diluted 1:10 with our smallest copy number between 2.2 and 3 copies per reaction. SHERLOCK detection reactions were run with three technical replicates per dilution factor. Fluorescence values for these technical replicates were background‐subtracted and averaged (± 1 *SD*) after 1 hr at 37°C.

The delta smelt gBlock dilution series was additionally subject to qPCR for comparison of assay sensitivity. Again, three technical replicates were analysed. The qPCRs were comprised of the following: gBlock template, 0.9 µm forward and reverse primer, 0.06 µm TaqMan Probe (Applied Biosystems), and 1 × TaqMan Universal PCR Master Mix (Applied Biosystems) for 40 cycles with a 10‐min initial denaturation at 95°C, 15 s cycle denaturation at 95°C and 1 min annealing at 63°C. Images were recorded after each cycle. Reactions were carried out in the same BioRad CFX96 Touch Real‐Time PCR Detection System used in SHERLOCK detection reactions. BioRad CFX Maestro Software was used to obtain relative fluorescence units for each sample. Duplicate negative control samples were included on each plate and their average was used to background‐subtract all samples for each cycle. Fluorescence for these technical replicates were background‐subtracted and averaged (± 1 *SD*) after 1 hr at 37°C.

### 2.7 Time course reactions for speed comparison

We assessed the relative magnitude of background‐subtracted fluorescence signal over time for each of the three osmerid SHERLOCK assays. Species‐specific crRNA was combined with 10 ng DNA extracted from tissue for each target species, with three technical replicates per species. Fluorescence was measured every 5 min over a 110‐min time‐course and then background‐subtracted. The technical replicates were averaged per species (± 1 *SD*).

We also compared the delta smelt SHERLOCK assay results mentioned in the previous paragraph with the speed of a TaqMan qPCR assay, which amplifies the same target region of *cyt‐b*. The starting DNA template (10 ng) was from the same sample as used for the SHERLOCK reaction, with three technical replicates. The qPCR conditions were the same as described above for the gBlock qPCRs. Fluorescence was measured every 5 min over a 50‐min time‐course and then background‐subtracted. The technical replicates were averaged (± 1 *SD*).

### 2.8 Mucus swabbing

We performed swabbing experiments on hatchery delta smelt from the UC Davis Fish Conservation and Culture Laboratory (FCCL). Individual fish mucus was collected using a Puritan Rayon swab wiped along the lateral line of the body from head to tail five times. Swabs were then swirled in either phosphate‐buffered saline (PBS) or Qiagen ATL cell lysis buffer (buffer ATL), depending on the method used.

Three types of nonextraction methods were attempted: mucus swabs in 50 µl 1 × PBS buffer, mucus swabs in 300 µl of 1 × PBS buffer and mucus swabs in 300 µl of buffer ATL. The human saliva DNA extraction centrifugation technique described in Gootenberg et al. (2017) was evaluated with the modification that the mucus swab was placed in 500 µl of 1 × PBS buffer instead of 400 µl. Lastly, a traditional DNA extraction protocol for swabs using the QIAamp MiniPrep kit (Qiagen) was utilized following the manufacturer's instructions. Ten biological replicates were collected for each of the five tests.

Wild smelt were swabbed to determine if results were similar to those obtained from swabbing hatchery fish. Because wild delta smelt are rare and protected, wild wakasagi were caught and swabbed in the SFE (Cache Slough and Liberty Island, Lower Sacramento Ship Channel, and Suisun Marsh) as a surrogate species. All wakasagi swabs were swirled in 300 µl PBS buffer directly after collection and frozen until the SHERLOCK detection reaction was prepared in the laboratory. Two “no template” negative controls and two positive tissue DNA controls were run on each plate. Reactions were incubated at 37°C for 1 hr unless otherwise indicated. Additionally, qPCR was performed on all biological replicates of extraction‐free mucus swabs in 300 µl 1 × PBS for delta smelt. The qPCRs and analysis methods were the same as described above for the gBlock qPCR.

### 2.9 Lateral flow detection reactions

SHERLOCK‐Cas13a reactions were additionally detected using commercially available lateral flow strips (Milenia HybriDetect 1, TwistDx). Lateral flow visualization was achieved by substituting a custom IDT FAM‐Biotin reporter (Myhrvold et al., 2018) (/56‐FAM/UUUUUUUUUUUUUU/3Bio) in place of RNase Alert at a final concentration of 1 µm. The 22‐µl reactions were incubated at 37°C for 1 hr, unless otherwise indicated, and 20 µl was diluted 1:4 or 1:5 in HybriDetect 1 Assay Buffer. Lateral flow strips were then inserted into the wells containing diluted reactions and incubated at room temperature for 5 min. After incubation, strips were removed and RGB images were collected using a smartphone. Lateral flow band intensity was quantified using Fiji imaging software (Schindelin et al., 2012). RGB images were converted to 32‐bit greyscale images. A vertical straight line (“line” tool) was drawn through the centre of the band (this avoided artefacts related to band fading on both sides of the flow strip). Greyscale values along the line were determined using the “Analyze ‐ plot profile” tool and the minimum intensity value (i.e., darkest pixel) was recorded and used as the intensity value of the band. The background pixel intensities were averaged and the band intensity was subtracted to yield the adjusted band intensity.

For each smelt assay, a comparison of extracted genomic DNA from tissue (50 ng/µl), gBlock (diluted to 10–14 thousand copies per microlitre), nonextracted DNA mucus swabs (in 300 µl 1 × PBS buffer), genomic DNA from an off‐target species (wakasagi for both the delta smelt and longfin smelt assays and delta smelt for the wakasagi assay) and a negative control were tested. Additionally, a lateral flow time series experiment using nonextracted delta smelt mucus stored in 300 µl of 1 × PBS was used to determine the speed of positive detection.

## 3 RESULTS

### 3.1 Specificity and sensitivity using tissue and synthetic oligos

Using DNA extracted from tissue, we assayed 40 samples of each target osmerid species along with 20 samples of each nontarget osmerid species using species‐specific crRNAs. All individuals amplified for their species‐specific assay and no individuals cross‐amplified for either of the other two nontarget species assays (Figure 1c). Additionally, we confirmed that 24 other fish species commonly found in the SFE did not produce false positive SHERLOCK results for any of the assays (Figure 1d; Table S4), further validating 100% species specificity. We tested the sensitivity of our three osmerid SHERLOCK assays using synthetic gBlock oligonucleotide DNA fragments (Table S5). The DSM and LFS assays could reliably detect their respective DNA targets down to ~ 300 copies per reaction, whereas the WAG assay sensitivity was slightly lower (~2,000 copies per reaction) (Figure 1e). These limits of detection should be effective for reliably detecting the mitochondrially encoded *cyt‐b* target even when DNA concentrations are low, as mitochondrial DNA copy number varies across species and tissue types but typically ranges from hundreds to thousands of copies per cell in eukaryotes (Cole, 2016).

### 3.2 Speed and sensitivity comparisons with qPCR

We next determined how rapidly positive detections could be made after initiating the SHERLOCK reaction by reading fluorescence every 5 min for a total of 1 hr (Figure 1f). For all three assays, the minimum positive detection time was less than 20 min for 20 ng of input DNA, and reached a maximum at ~ 30 min, remaining stable for the remainder of the time‐course (Figure 1f). When directly comparing SHERLOCK and qPCR assays for DSM (with both sets of primers and probes targeting the same *cyt‐b* region), SHERLOCK detections were ~ 2.5 times more rapid when using the same fluorescence reader and averaged an absolute signal intensity that was 15 times higher, although this may be influenced by the amount of reporter in each assay (Figure 1g; Figure S3a). However, the DSM qPCR assay was more sensitive than SHERLOCK as tested on synthetic gBlock oligo DNAs (Figure S3b). The qPCR limit of detection was ~ 3 copies per reaction in comparison to ~ 300 copies per reaction with SHERLOCK. This decreased sensitivity is probably due to conducting a SHERLOCK one‐pot reaction (combining RPA and Cas13a detection in a single reaction for increased speed and convenience) versus conducting a two‐step SHERLOCK protocol, which is more sensitive and typically capable of single molecule detection (Kellner, Koob, Gootenberg, Abudayyeh, & Zhang, 2019). Serial dilutions of the delta smelt synthetic oligo showed that minimum positive detection time was 20 min or less when input DNA was ~ 3,000 copies per reaction or greater (Figure S4).

### 3.3 Optimization of minimally invasive sampling

**Figure 2:**
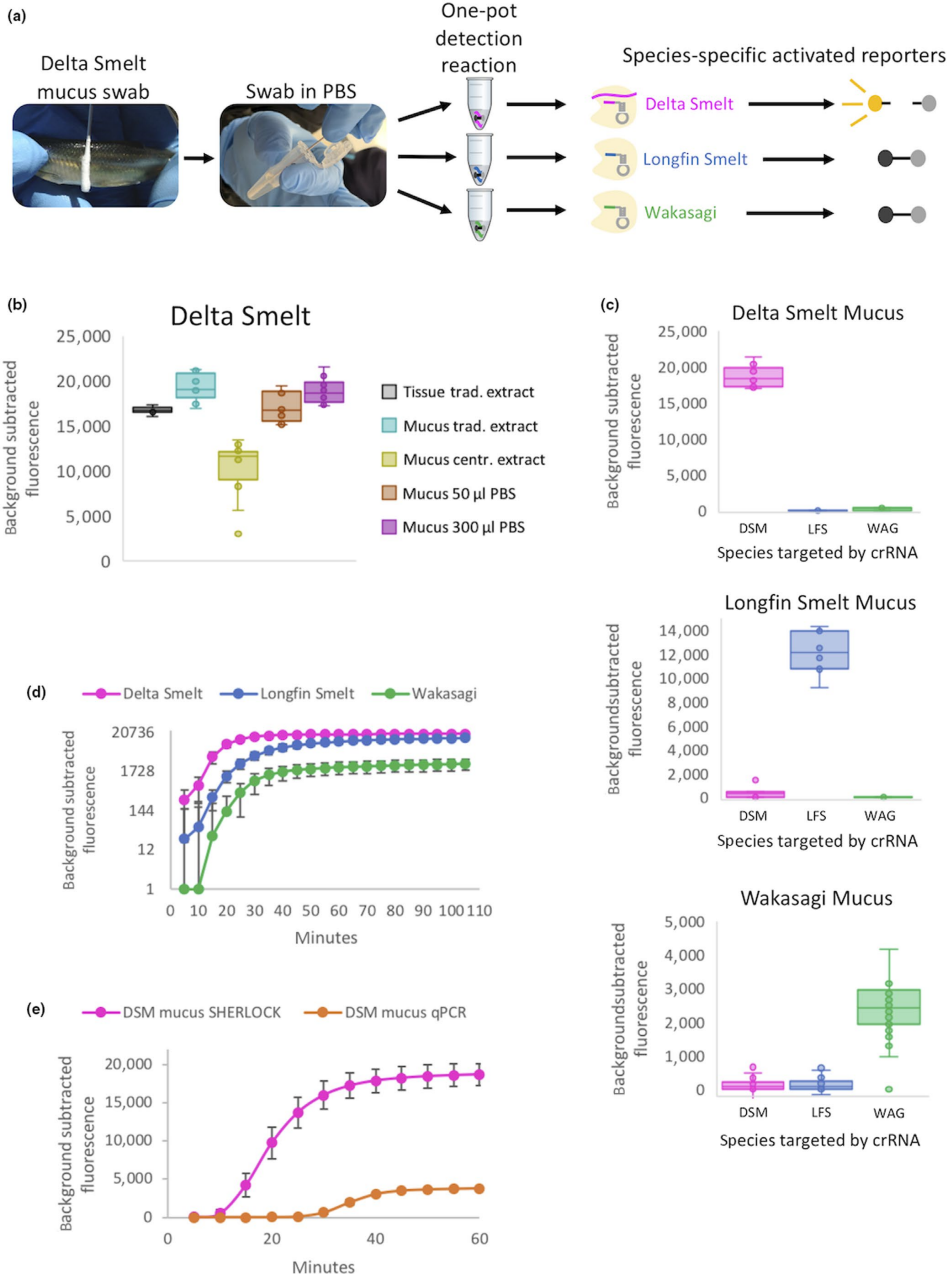
Characterization of SHERLOCK assays using noninvasive mucus swabs. (a) Schematic of a rapid and noninvasive species detection method using mucus swabs (delta smelt shown here as an example) placed directly in PBS followed with three one‐pot SHERLOCK reactions (one for each smelt assay). Only a reaction containing crRNA specific to delta smelt will fluoresce. (b) Evaluation of different methods for noninvasive species detection compared to DNA extracted from tissue. Mucus swabs were used both with and without DNA extraction and with varying volumes of PBS. Delta smelt caudal fin tissue or mucus swabs were used and SHERLOCK fluorescence was measured after 1 hr. Median and interquartile range are shown for each boxplot. *N* = 6 for positive control (DNA extracted from tissue) and *N* = 10 for all other methods. Trad. = traditional; centr. = centrifugation (see Methods). (c) Species specificity for each osmerid assay demonstrated by SHERLOCK fluorescence after 1 hr. For each target species, mucus swabs placed directly in 300 µl PBS were tested against all three species‐specific crRNAs. Boxplots display median and interquartile range for each DNA and crRNA assay combination. DSM = delta smelt, LFS = longfin smelt, WAG = wakasagi. Median and interquartile range are shown for each boxplot. For target species, *N* = 10 (DSM), *N* = 7 (LFS) and *N* = 39 (WAG) and ranged from three to 10 for each nontarget species. (d) Rapid detection of SHERLOCK fluorescence for mucus swabs placed directly in 300 µl PBS from each target species. Fluorescence was measured every 5 min over a 110‐min time‐course. Average fluorescence values were plotted with error bars = 1 *SD*. DSM: *N* = 10; LFS: *N* = 7; WAG: *N* = 39. (e) Time comparison of SHERLOCK and qPCR assays using delta smelt mucus swabs and targeting the same locus. SHERLOCK conditions and data are as in (d). Fluorescence was measured every 5 min over a 60‐min time‐course. Average values for *N* = 10 are plotted for each assay with 1 *SD* error bars.

Once the specificity, sensitivity and speed of SHERLOCK results were characterized for traditionally extracted tissue, we focused on developing a method for accessing the target species’ DNA with minimal invasiveness and requiring little to no additional upstream procedures prior to commencing the SHERLOCK reaction. Fish mucus, which is abundant and covers all epithelial surfaces, can be swabbed with a brush to obtain DNA samples, and this method has been successfully used for genotyping and high‐throughput sequencing (Taslima, Davie, McAndrew, & Penman, 2016; Taslima, Taggart, Wehner, McAndrew, & Penman, 2017). More generally, mucus swabbing is used for genetic analysis of many other diverse organisms including humans (Clarke et al., 2014), amphibians (Pidancier, Miquel, & Miaud, 2003) and molluscs (Henley, Grobler, & Neves, 2006). We first tested both DNA extraction‐ and nonextraction‐based methods for performing SHERLOCK on mucus swabs from delta smelt (Figure 2a). We observed that even without DNA extraction, by simply swirling the swabs in tubes containing PBS, we could detect robust SHERLOCK fluorescence, comparable in magnitude to DNA extracted from fin tissue (Figure 2b). We proceeded to test this noninvasive approach in all three osmerid SHERLOCK assays, and found that the mucus swabbing in PBS method performed well across all assays and displayed a high degree of species‐specificity, considerably reducing processing time and making it ideal for field applications (Figure 2c). Furthermore, similar to DNA extracted from tissue, SHERLOCK fluorescence could be detected 15–20 min after reaction commencement (Figure 2d,e) and is approximately twice as rapid as qPCR (Figure 2e), providing additional speed for field deployability and time‐sensitive applications.

### 3.4 Enabling genetic assay field deployment

**Figure 3:**
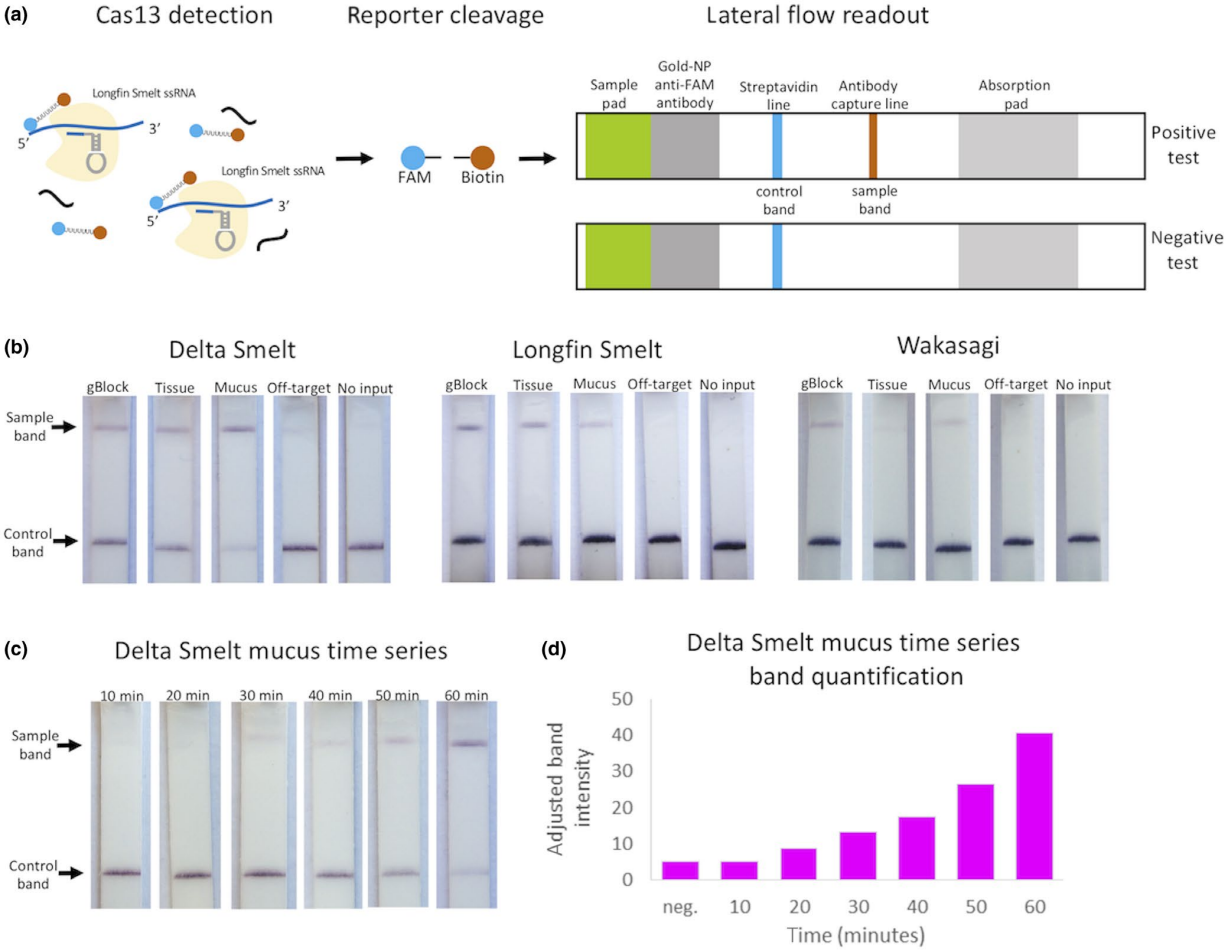
Lateral flow detection of extracted DNA from tissue as well as nonextracted mucus swabs. (a) Schematic of SHERLOCK instrument‐free detection using a FAM‐ and biotin‐labelled RNA oligonucleotide reporter and commercial lateral flow strips. Uncleaved reporter accumulates as anti‐FAM antibody/gold nanoparticle conjugates at the control (Streptavidin) line. If target DNA is present, the reporter is cleaved by Cas13a, resulting in conjugate binding at the antibody capture line. (b) For each species‐specific assay, on‐target synthetic gBlock, tissue DNA and mucus swabs (in 300 µl PBS) were detected with lateral flow. Off‐target tissue DNA (see Methods for species used) and no‐template reactions were included as negative controls. (c) Time‐course of delta smelt mucus swab detection over 60 min. (d) Quantification of band intensities from (c). Neg., no‐template control.

As the SHERLOCK fluorescence assay performed well, with high specificity and rapidity, using a minimally invasive swabbing technique without the need for DNA extractions, we moved forward with refinements that could further aid field deployment. We tested a visual, equipment‐free SHERLOCK readout method using lateral flow strips and dual labelled RNA reporter (Gootenberg et al., 2017, 2018) (Figure 3a). When conducting the SHERLOCK lateral flow assay for DNA extracted from tissue, mucus swabs in PBS and synthetic template all showed positive visual bands for each species‐specific assay tested 1 hr after the reaction start (Figure 3b). The positive bands for wakasagi were noticeably lighter, probably due to the reduced signal strength of that assay, as seen for detection of both tissue and mucus fluorescence (Figures 1f and 2d). Based on the successful visual read‐outs from the genetic identification at 1 hr, we conducted a time series to determine the minimum time needed to visually detect a positive band for delta smelt mucus with the naked eye. Using technical replicates, we reliably saw bands at 40 min, with occasional detection at 30 min (see Figure 3c for representative strips at each time point), approximately twice the time needed to conduct genetic identification using a fluorescence reader. The 15–20‐min loss in speed, and probably some loss in sensitivity, will need to be considered against the ease of equipment‐free detection. Additionally, results will vary depending on each crRNA assay, because some have stronger signals than others, and each mucus swab, because they can pick up varying amounts of DNA from the target individual.

## 4 DISCUSSION

The adaptation of the SHERLOCK method described in this study has all the necessary attributes for full field deployment. The DNA extraction‐free direct use of mucus swabs is a significant advancement that allows all subsequent steps to be performed outside the laboratory. Because the isothermal RPA amplification requires only a constant low temperature (Piepenburg, Williams, Stemple, & Armes, 2006) and the entire reaction can occur in a single tube with lyophilized reagents (Gootenberg et al., 2017), SHERLOCK can easily be performed in the field. The reaction can occur in the palm of the hand (and subsequently be put onto a lateral flow strip) or in a small portable device with temperature control and fluorescence detection (e.g., ESEQuant TS2, Qiagen). The speed of detection (<20 min when using a fluorescence device), along with the option of instrument‐free detection, are also critical components for field usage. In comparison, hand‐held qPCR instruments such as Biomeme's Franklin unit takes 30–60 min to detect target DNA (biomeme.com) and as a qPCR platform may be less sensitive than SHERLOCK. It is anticipated that the entire protocol, from obtaining a sample to genetic identification, could be completed in less than 1 hr, which enables near real‐time species diagnostics. Altogether, our results provide an important proof‐of‐concept that SHERLOCK can be reliably used in a variety of ecological and environmental monitoring settings to obtain accurate, sensitive and rapid genetic results. Future studies may expand its use to other organisms and finer‐scale taxonomic differentiation, such as discriminating between subspecies. CRISPR‐based methods can also be used for detecting specific organisms in environmental DNA samples (Williams et al., 2019). As a whole, SHERLOCK and other CRISPR methods such as DETECTR (Chen et al., 2018) and FLASH (Quan et al., 2019) have the potential for widespread application in ecology due to their sensitivity, accuracy and speed. By embracing CRISPR methods, ecology and conservation biology will be able to bring rapid, genetic‐based taxonomic identification to the most remote field settings. Furthermore, the ease of use of SHERLOCK and similar assays will expand the power of CRISPR beyond the realm of geneticists and move it into the hands of field biologists, unlocking the potential of this transformative technology to redefine how, where and by whom genetic identification occurs in the future.

## CONFLICT OF INTERESTS

J.S.G., O.O.A. and F.Z. are cofounders of Sherlock Biosciences. F.Z. is a cofounder and advisor for Beam Therapeutics, Editas Medicine, Pairwise Plants, and Arbor Biotechnologies. J.S.G. and O.O.A. are advisors for Beam Therapeutics. J.S.G. is a campus advisor of Benchling, Inc.

## AUTHOR CONTRIBUTIONS

M.R.B. and R.P.N. developed the initial concept; A.M.G., J.S.G., O.O.A. and A.M.S. provided input on conceptual design; A.M.G. developed the techniques and performed the experiments with input from M.R.B., R.P.N., J.S.G., O.O.A., F.Z. and A.M.S.; M.R.B. and R.P.N. conducted data analysis; M.R.B., A.M.G. and R.P.N. wrote the manuscript. All authors discussed the results and provided edits and approval of the manuscript.

## Supporting information

Supplementary Material

## Data Availability

All data generated in this study have been made available via Data Dryad: Baerwald, Melinda et al. (2019), Rapid and accurate species identification for ecological studies and monitoring using CRISPR‐based SHERLOCK, Dryad, Dataset, https://doi.org/10.5061/dryad.hdr7sqvd3

## References

[men13186-bib-0001] Abudayyeh, O. O. , Gootenberg, J. S. , Kellner, M. J. , & Zhang, F. (2019). Nucleic acid detection of plant genes using CRISPR‐Cas13. The CRISPR Journal, 2(3), 165–171. 10.1089/crispr.2019.0011 31225754 PMC7001462

[men13186-bib-0003] Baerwald, M. R. , Schumer, G. , Schreier, B. M. , & May, B. (2011). TaqMan assays for the genetic identification of delta smelt (Hypomesus transpacificus) and wakasagi smelt (Hypomesus nipponensis). Molecular Ecology Resources, 11(5), 784–785. 10.1111/j.1755-0998.2011.03011.x 21443553

[men13186-bib-0004] Benjamin, A. , Sağlam, İ. K. , Mahardja, B. , Hobbs, J. , Hung, T.‐C. , & Finger, A. J. (2018). Use of single nucleotide polymorphisms identifies backcrossing and species misidentifications among three San Francisco estuary osmerids. Conservation Genetics, 19(3), 701–712. 10.1007/s10592-018-1048-9

[men13186-bib-0005] Brandl, S. , Schumer, G. , Schreier, B. M. , Conrad, J. L. , May, B. , & Baerwald, M. R. (2015). Ten real‐time PCR assays for detection of fish predation at the community level in the San Francisco Estuary‐Delta. Molecular Ecology Resources, 15(2), 278–284. 10.1111/1755-0998.12305. 25042458

[men13186-bib-0006] CDFW (California Department of Fish and Wildlife) (2019). State and federally listed endangered and threatened animals of California. Retrieved from https://nrm.dfg.ca.gov/FileHandler.ashx?DocumentID=109405&inline.

[men13186-bib-0007] Chen, J. S. , Ma, E. , Harrington, L. B. , Da Costa, M. , Tian, X. , Palefsky, J. M. , & Doudna, J. A. (2018). CRISPR‐Cas12a target binding unleashes indiscriminate single‐stranded DNase activity. Science (New York, N.Y.), 360(6387), 436–439. 10.1126/science.aar6245 PMC6628903 29449511

[men13186-bib-0008] Clarke, A. C. , Prost, S. , Stanton, J.‐A.‐ L. , White, W. T. J. , Kaplan, M. E. , & Matisoo‐Smith, E. A. (2014). From cheek swabs to consensus sequences: An A to Z protocol for high‐throughput DNA sequencing of complete human mitochondrial genomes. BMC Genomics, 15(1), 68 10.1186/1471-2164-15-68 24460871 PMC3922791

[men13186-bib-0009] Cole, L. W. (2016). August 18). The evolution of per‐cell organelle number. *Frontiers in Cell and* . Developmental Biology, 4(), 85–. 10.3389/fcell.2016.00085 PMC4988970 27588285

[men13186-bib-0010] R Core Team (2019). R: A language and environment for statistical computing. R Core Team . Retrieved from https://www.r‐project.org/.

[men13186-bib-0011] Gootenberg, J. S. , Abudayyeh, O. O. , Kellner, M. J. , Joung, J. , Collins, J. J. , & Zhang, F. (2018). Multiplexed and portable nucleic acid detection platform with Cas13, Cas12a, and Csm6. Science (New York, N.Y.), 360(6387), 439–444. 10.1126/science.aaq0179 PMC5961727 29449508

[men13186-bib-0012] Gootenberg, J. S. , Abudayyeh, O. O. , Lee, J. W. , Essletzbichler, P. , Dy, A. J. , Joung, J. , … Zhang, F. (2017). Nucleic acid detection with CRISPR‐Cas13a/C2c2. Science (New York, N.Y.), 356(6336), 438–442. 10.1126/science.aam9321 PMC5526198 28408723

[men13186-bib-0013] Henley, W. , Grobler, P. , & Neves, R. (2006). Non‐invasive method to obtain DNA from freshwater mussels (Bivalvia: Unionidae). Journal of Shellfish Research, 25(3), 975–977.

[men13186-bib-0014] Hubert, N. , Hanner, R. , Holm, E. , Mandrak, N. E. , Taylor, E. , Burridge, M. , … Bernatchez, L. (2008). Identifying Canadian freshwater fishes through DNA barcodes. PLoS One, 3(6), e2490 10.1371/journal.pone.0002490 22423312 PMC3278308

[men13186-bib-0015] Kellner, M. J. , Koob, J. G. , Gootenberg, J. S. , Abudayyeh, O. O. , & Zhang, F. (2019). SHERLOCK: Nucleic acid detection with CRISPR nucleases. Nature Protocols, 14(10), 2986–3012. 10.1038/s41596-019-0210-2 31548639 PMC6956564

[men13186-bib-0016] Knott, G. J. , & Doudna, J. A. (2018). CRISPR‐Cas guides the future of genetic engineering. Science, 361(6405), 866–869. 10.1126/science.aat5011 30166482 PMC6455913

[men13186-bib-0017] Koressaar, T. , & Remm, M. (2007). Enhancements and modifications of primer design program Primer3. Bioinformatics, 23(10), 1289–1291. 10.1093/bioinformatics/btm091 17379693

[men13186-bib-0018] Kumar, S. , Stecher, G. , & Tamura, K. (2016). MEGA7: Molecular evolutionary genetics analysis version 7.0 for bigger datasets. Molecular Biology and Evolution, 33(7), 1870–1874. 10.1093/molbev/msw054 27004904 PMC8210823

[men13186-bib-0019] Myhrvold, C. , Freije, C. A. , Gootenberg, J. S. , Abudayyeh, O. O. , Metsky, H. C. , Durbin, A. F. , … Sabeti, P. C. (2018). Field‐deployable viral diagnostics using CRISPR‐Cas13. Science, 360(6387), 444–448. 10.1126/science.aas8836 29700266 PMC6197056

[men13186-bib-0020] Pidancier, N. , Miquel, C. , & Miaud, C. (2003). Buccal swabs as a non‐destructive tissue sampling method for DNA analysis in amphibians. Herpetological Journal, 13(4), 175–178.

[men13186-bib-0021] Piepenburg, O. , Williams, C. H. , Stemple, D. L. , & Armes, N. A. (2006). DNA detection using recombination proteins. PLoS Biology, 4(7), e204 10.1371/journal.pbio.0040204 16756388 PMC1475771

[men13186-bib-0022] Pinzón, J. H. , Sampayo, E. , Cox, E. , Chauka, L. J. , Chen, C. A. , Voolstra, C. R. , & Lajeunesse, T. C. (2013). Blind to morphology: Genetics identifies several widespread ecologically common species and few endemics among Indo‐Pacific cauliflower corals (Pocillopora, Scleractinia). Journal of Biogeography, 40(8), 1595–1608. 10.1111/jbi.12110

[men13186-bib-0023] Quan, J. , Langelier, C. , Kuchta, A. , Batson, J. , Teyssier, N. , Lyden, A. , … Crawford, E. D. (2019). FLASH: A next‐generation CRISPR diagnostic for multiplexed detection of antimicrobial resistance sequences. Nucleic Acids Research, 47(14), e83–e83. 10.1093/nar/gkz418 31114866 PMC6698650

[men13186-bib-0024] Schindelin, J. , Arganda‐Carreras, I. , Frise, E. , Kaynig, V. , Longair, M. , Pietzsch, T. , … Cardona, A. (2012). Fiji: An open‐source platform for biological‐image analysis. Nature Methods, 9, 676–682. 10.1038/nmeth.2019 22743772 PMC3855844

[men13186-bib-0025] Taslima, K. , Davie, A. , McAndrew, B. J. , & Penman, D. J. (2016). DNA sampling from mucus in the Nile tilapia, *Oreochromis niloticus* : Minimally invasive sampling for aquaculture‐related genetics research. Aquaculture Research, 47(12), 4032–4037. 10.1111/are.12809

[men13186-bib-0026] Taslima, K. , Taggart, J. B. , Wehner, S. , McAndrew, B. J. , & Penman, D. J. (2017). Suitability of DNA sampled from Nile tilapia skin mucus swabs as a template for ddRAD‐based studies. Conservation Genetics Resources, 9(1), 39–42. 10.1007/s12686-016-0614-z

[men13186-bib-0027] Untergasser, A. , Cutcutache, I. , Koressaar, T. , Ye, J. , Faircloth, B. C. , Remm, M. , & Rozen, S. G. (2012). Primer3‐new capabilities and interfaces. Nucleic Acids Research, 40(15), e115–e115. 10.1093/nar/gks596 22730293 PMC3424584

[men13186-bib-0028] USFWS (1993). Endangered and threatened wildlife and plants: determination of threatened status for the Delta Smelt. Federal Register, 53, (12854–12864).

[men13186-bib-0029] Vrijenhoek, R. C. (2009). Cryptic species, phenotypic plasticity, and complex life histories: Assessing deep‐sea faunal diversity with molecular markers. Deep Sea Research Part II: Topical Studies in Oceanography, 56(19–20), 1713–1723. 10.1016/J.DSR2.2009.05.016

[men13186-bib-0030] Williams, M.‐A. , O'Grady, J. , Ball, B. , Carlsson, J. , Eyto, E. , McGinnity, P. , … Parle‐McDermott, A. (2019). The application of CRISPR‐Cas for single species identification from environmental DNA. Molecular Ecology Resources, 19(5), 1106–1114. 10.1111/1755-0998.13045 31177615

