# Supplementary material for: Rapid and accurate species identification for ecological studies and monitoring using CRISPR‐based SHERLOCK
Source: Mol Ecol Resour. 2020 Jun 13;20(4):961–70. doi: 10.1111/1755-0998.13186 (PMC7497203; doi:10.1111/1755-0998.13186)
Supplement: Supplementary file 1 — Supplementary Material [file MEN-20-961-s001.pdf]

Supplementary Information

Rapid and accurate species identification for ecological studies and monitoring using CRISPR-based SHERLOCK

Melinda R. Baerwald[1*], Alisha M. Goodbla[2], Raman P. Nagarajan[2], Jonathan S. Gootenberg[3,4,5,6,7], Omar O. Abudayyeh[3,4,5,6,8], Feng Zhang[3,4,5,6], and Andrea M. Schreier[2]

[1] California Department of Water Resources, 3500 Industrial Blvd, Sacramento, CA 95691, USA.
[2] Department of Animal Science, University of California Davis, One Shields Ave, Davis, CA, USA.
[3] Broad Institute of the Massachusetts Institute of Technology (MIT) and Harvard, Cambridge, MA 02142, USA.
[4] McGovern Institute for Brain Research, MIT, Cambridge, MA 02139, USA.
[5] Department of Brain and Cognitive Science, MIT, Cambridge, MA 02139, USA.
[6] Department of Biological Engineering, MIT, Cambridge, MA 02139, USA.
[7] Department of Systems Biology, Harvard University, Boston, MA 02115, USA.
[8] Department of Health Sciences and Technology, MIT, Cambridge, MA 02139, USA.

* Corresponding author. Email: melinda.baerwald@water.ca.gov

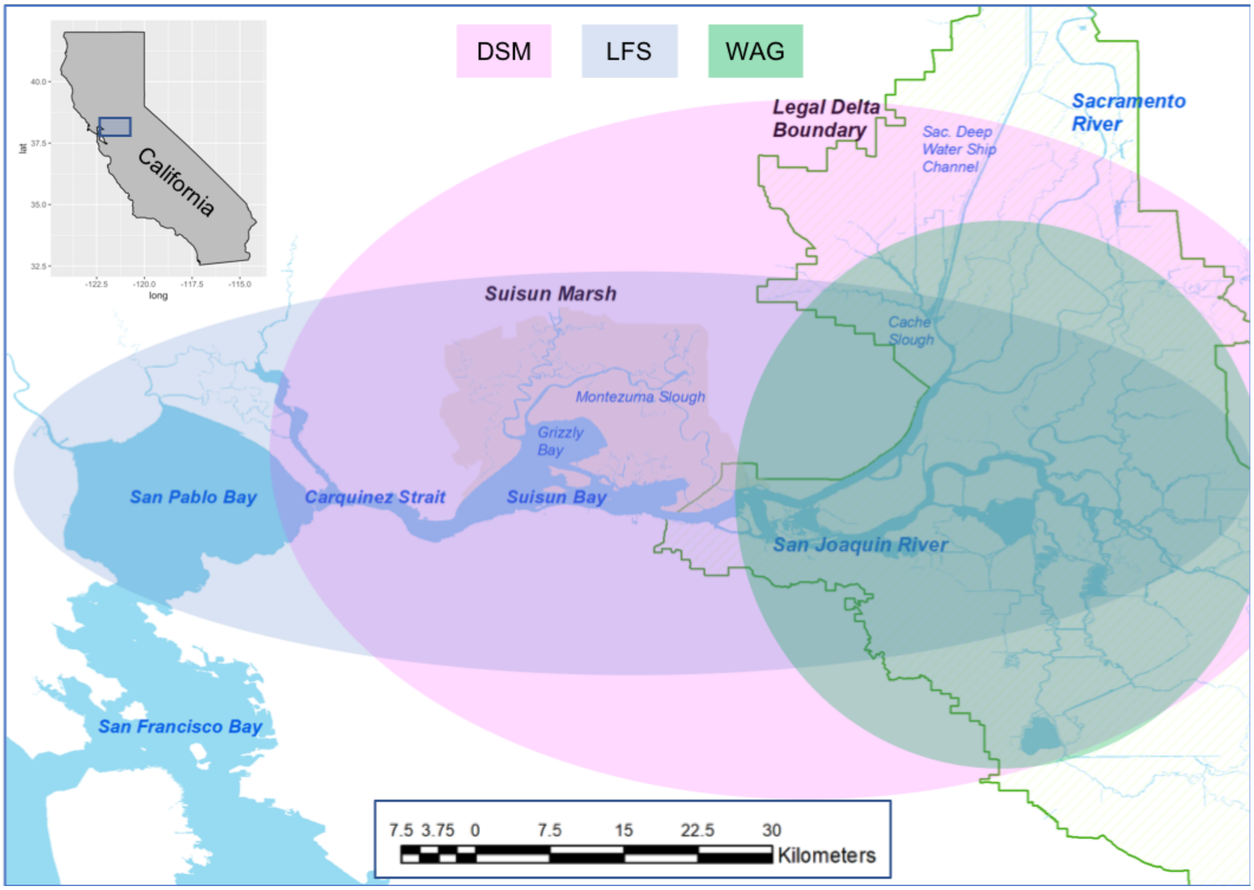

Supplementary Figure 1. Map of San Francisco Estuary, with general ranges of the three osmerid species highlighted. DSM, Delta Smelt; LFS, Longfin Smelt; WAG, Wakasagi.

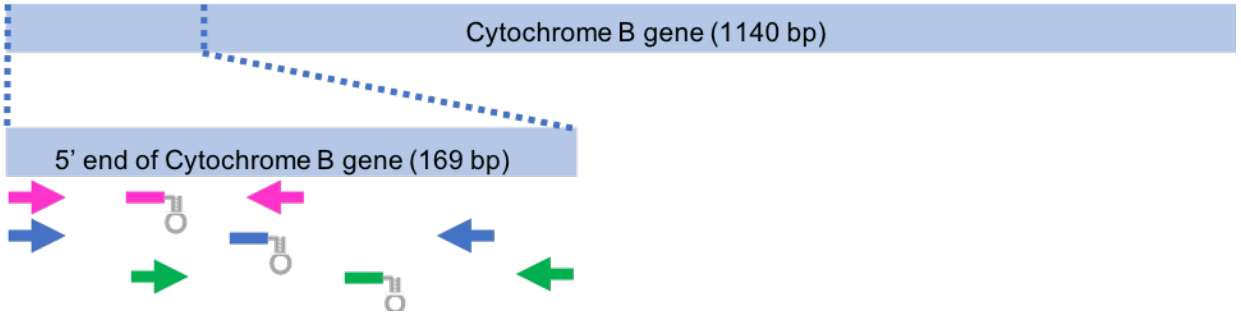

Supplementary Figure 2. Relative position of RPA primers and crRNA probes for three smelt species. Arrows signify RPA primers with crRNA probes shown between them.
Delta Smelt (magenta) primers amplify 85 bp, Longfin Smelt (blue) primers amplify 142 bp, and Wakasagi (green) primers amplify 135 bp.

a)

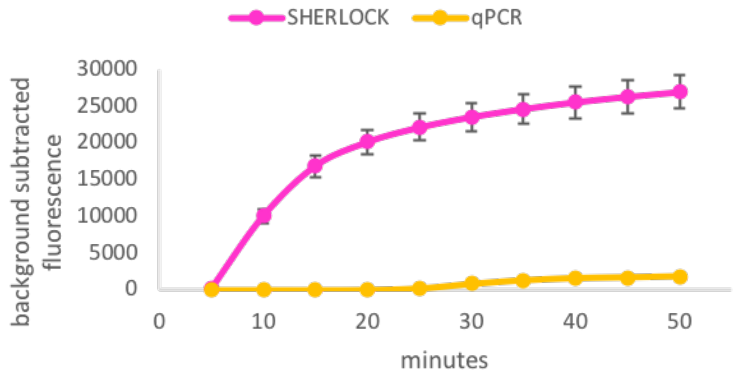

b)

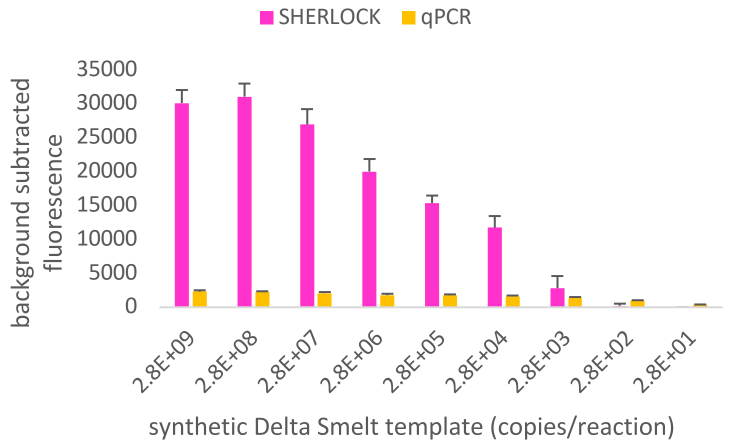

Supplementary Figure 3. Speed and sensitivity comparison of SHERLOCK and qPCR targeting the same Cyt-B region in Delta Smelt. a) Time course comparison between SHERLOCK and qPCR using 2.8 x 10^6 copies/reaction of synthetic Delta Smelt DNA (gBlock).. Fluorescence was measured every 5 minutes over a 50-minute time course. Three technical replicates were averaged ± 1 S.D. **b)** Limit of detection comparison between SHERLOCK assay using Delta Smelt specific crRNA and qPCR TaqMan assay using Delta Smelt specific probe. For both SHERLOCK and qPCR, serial dilutions of Delta Smelt DNA were derived from a synthetic gBlock template. Fluorescence was measured after one hour and bars represent means ± 1 S.D. from 3 technical replicates.

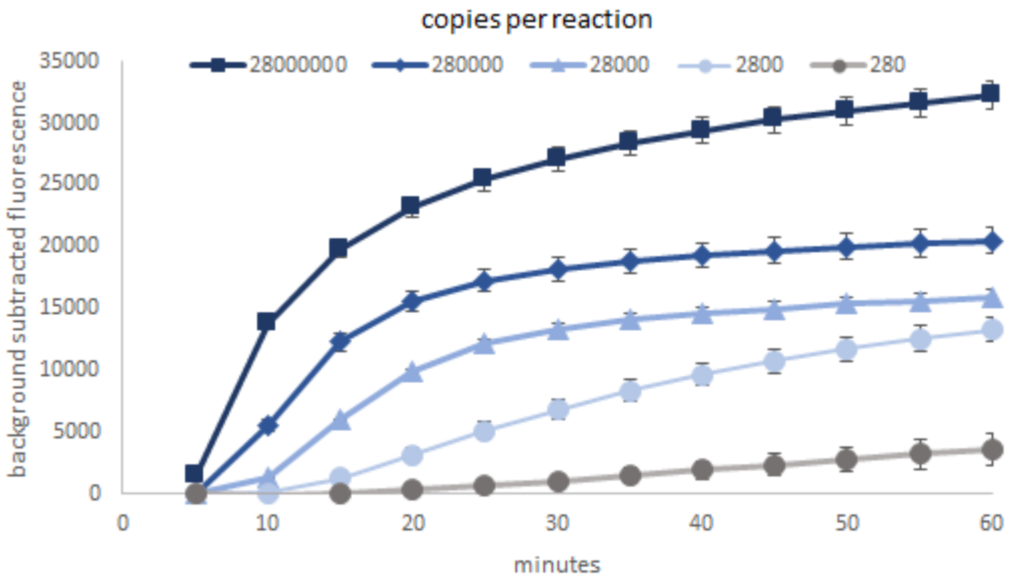

Supplementary Figure 4. Time course comparison of synthetic Delta Smelt DNA (gBlock), with copies per reaction ranging from 280 – 28,000,000. Fluorescence was measured every 5 minutes over a 60-minute time course. Three technical replicates were averaged ± 1 S.D.

Supplementary Table 1. RPA primer sequences used in study. Forward primers contain the T7 promoter sequence (aatcTAATACGACTCACTATAggg) at their 5' end, with primer DSM_LFS_F also containing an additional four random nucleotides upstream of this.

**RPA Primer Name**	**Sequence**	**Target Species**
DSM_LFS_F	AATCTAATACGACTCACTATAGGGAATGGCCAACCTTCGGAAA	Longfin Smelt, Delta Smelt
LFS_R1	CYGTRAGGATTTGRATRATRAGRCA	Longfin Smelt
DSM_R	GARATATTRGAGGGTGCAGG	Delta Smelt
WAG_F3	AATCTAATACGACTCACTATAGGGAAAATTACCAATGACGCCCTAGTT	Wakasagi
WAG_R3	CAGTATAGTGCATAGCCAAAA	Wakasagi

Supplementary Table 2. List of crRNA sequences used in this study. All three crRNAs contain the same direct repeat sequence (gggGAUUUAGACUACCCCAAAAACGAAGGGGACUAAAAC) at the 5' end. Spacer sequences are underlined. Diagnostic polymorphisms that distinguish the smelt species are bolded.

**Name**	**Target Species**	**Complete crRNA sequence**
WS_crRNA3	Wakasagi	GGGGAUUUAGACUACCCCAAAAACGAAGGGGACUAAAACCA**C**A**CC**CCAAG**A**AGGGA**U**CCAAAGUUUC
LFS_crRNA1	Longfin Smelt	GGGGAUUUAGACUACCCCAAAAACGAAGGGGACUAAAACGA**CG**CA**G**AUAUU**A**GAGGGUGCAGGUAAA
DS_crRNA3	Delta Smelt	GGGGAUUUAGACUACCCCAAAAACGAAGGGGACUAAAACAC**A**AC**A**GCGUC**G**UUGGUAAUUUU**C**AGGA

Supplementary Table 3. Target mitochondrial DNA used in this study.

**Species**	**Partial Cytochrome b Gene Sequence**	Genbank Accession
Delta Smelt	AATGGCCAACCTTCGGAAAACCCATCCCCTCCTGAAAATTACCAACGACGCTCTTGTTGATCTGCCTGCACCCTCCAATATTTC	HQ667171
Longfin Smelt	AATGGCCAACCTTCGGAAAACCCACCCCATCCTAAAAATTACCAATGACGCCCTAGTTGATTTACCTGCACCCTCTAATATCTCCGTCTGATGAAACTTTGGCTCCCTCCTTGGGCTCTGCCTCATCATCCAAATCCTCACGG	KF013249
Wakasagi	AAAATTACCAACGACGCTCTTGTTGATCTGCCTGCACCCTCCAATATTTCTATCTGATGAAACTTTGGCTCCCTCCTTGGACTATGTCTTATTATTCAAATCCTCACAGGCCTATTCCTAGCCATGCACTACACTG	HQ667170

Supplementary Table 4. List of 27 fish species (common and Latin names) tested for cross-amplification using the 3 osmerid SHERLOCK assays. All of the species are commonly observed in the San Francisco Estuary.

**Common name**	**Latin name**
American Shad	*Alosa sapidissima*
Bigscale Logperch	*Percina macrolepida*
Bluegill Sunfish	*Lepomis macrochirus*
Channel Catfish	*Ictalurus punctatus*
Chinook Salmon	*Oncorhynchus tshawytscha*
Common Carp	*Cyprinus carpio*
Delta Smelt	*Hypomesus transpacificus*
Fathead Minnow	*Pimephales promelas*
Golden Shiner	*Notemigonus crysoleucas*
Green Sturgeon	*Acipenser medirostris*
Largemouth Bass	*Micropterus salmoides*
Longfin Smelt	*Spirinchus thaleichthys*
Mississippi Silverside	*Menidia beryllina*
Mosquitofish	*Gambusia affinis*
Prickley Sculpin	*Cottus asper*
Rainbow Trout	*Oncorhynchus mykiss*
Sacramento Splittail	*Pogonichthys macrolepidotus*
Shimofuri Goby	*Tridentiger bifasciatus*
Three-spined Stickleback	*Gasterosteus aculeatus*
Striped Bass	*Morone saxatilis*
Threadfin Shad	*Dorosoma petenense*
Tule Perch	*Hysterocarpus traskii*
Wakasagi	*Hypomesus nipponensis*
Warmouth	*Lepomis gulosus*
White Catfish	*Ameiurus catus*
White Sturgeon	*Acipenser transmontanus*
Yellowfin Goby	*Acanthogobius flavimanus*

Supplementary Table 5. Synthetic target sequences used in this study. Double-stranded DNA molecules (IDT gBlocks) contained a T7 promoter primer sequence (XXXXXTAATACGACTCACTATAggg) at their 5' end to enable transcription. A 20 base pair extension was added to the 5' and 3' ends to increase primer binding affinity.

**Species**	**Synthetic Gene Fragments**
Delta Smelt	AAATCTAATACGACTCACTATAGGGAATTCAACTACAAGAACCCTAATGGCCAACCTTCGGAAAACCCATCCCCTCCTGAAAATTACCAACGACGCTCTTGTTGATCTGCCTGCACCCTCCAATATTTCTATCTGATGAAACTTTGGCT
Longfin Smelt	AATTCTAATACGACTCACTATAGGGAATTCAACTACAAGAACCTTAATGGCCAACCTTCGGAAAACCCACCCCATCCTAAAAATTACCAATGACGCCCTACTTGATTTACCTGCACCCTCTAATATCTCCGTCTGATGAAACTTTGGCTCCCTCCTTGGGCTCTGCCTCATCATCCAAATCCTCACGGGCCTATTTCTAGCCATGCACT
Wakasagi	AATTCTAATACGACTCACTATAGGGCGGAAAACCCACCCCCTCCTAAAAATTACCAATGACGCCCTAGTTGATTTACCTGCACCCTCCAATATTTCAATCTGATGAAACTTTGGATCCCTTCTTGGGCTGTGTCTTATTATCCAAATCCTTACGGGCCTCTTTTTGGCTATGCACTATACTGCTGAGACTGCTACCGCTTTT
